# Mealtime Behaviors and Food Preferences of Students with Autism Spectrum Disorder

**DOI:** 10.3390/foods10010049

**Published:** 2020-12-26

**Authors:** Hae Jin Park, Su Jin Choi, Yuri Kim, Mi Sook Cho, Yu-Ri Kim, Ji Eun Oh

**Affiliations:** 1 Department of Nutritional Science & Food Management, Ewha Womans University, Seoul 03760, Korea; big85@ewhain.net (H.J.P.); sujin-choi@ewhain.net (S.J.C.); yuri.kim@ewha.ac.kr (Y.K.); misocho@ewha.ac.kr (M.S.C.); 2 Department of Special Education, Ewha Womans University, Seoul 03760, Korea; yuri1023@ewha.ac.kr; 3 College of Science & Industry Convergence, Ewha Womans University, Seoul 03760, Korea

**Keywords:** autism spectrum disorder, mealtime behaviors, food preferences

## Abstract

Autism spectrum disorder (ASD) is a neurodevelopmental disorder characterized by a lack of social communication and restrictive, repetitive behaviors or interests. This study aimed to examine the mealtime behaviors and food preferences of students with ASD. An online questionnaire on mealtime behavior and food preferences of ASD students was conducted by caregivers including parents, and the average age of ASD students was 14.1 ± 6.1. The analysis of mealtime behavior resulted in classification into three clusters: cluster 1, the “low-level problematic mealtime behavior group”; cluster 2, the “mid-level problematic mealtime behavior group”; and cluster 3, the “high-level problematic mealtime behavior group”. Cluster 1 included older students than other clusters and their own specific dietary rituals. Meanwhile, cluster 3 included younger students than other clusters, high-level problematic mealtime behavior, and a low preference for food. In particular, there were significant differences in age and food preference for each subdivided ASD group according to their eating behaviors. Therefore, the content and method of nutrition education for ASD students’ needs a detailed approach according to the characteristics of each group.

## 1. Introduction

The term “autism”, first introduced by Leo Kanner in 1943 [1], comes from the Greek word “autos”, which means “sel“ and “withi“ [2], and is one of the developmental disorders named for being in a state like “living in their own worlds” [1]. The prevalence of autism spectrum disorders is increasing worldwide [3,4,5]. According to 2016 Centers for Disease Control (CDC) data, about 1 in 54 children was estimated to have an autism spectrum disorder [6], and in Korea, the autism prevalence rate was reported to be 2.64% [7]. Autism spectrum disorder (ASD) is characterized by the two core areas of lack of social communication and restrictive, repetitive behaviors and interests, which impede the performance of roles in society and everyday life [8].

Children with ASD have a variety of problems related to feeding behaviors due to disorder characteristics, including lack of communication skills, social engagement, behavioral flexibility, sensory sensitivity, and limitations in range of areas of interest [9,10,11,12]. It has been reported that feeding problems are one of the characteristics of ASD and that there is a possible association between ASD children and problems related to mealtime [1,13]. In previous studies, the prevalence of problem feeding behavior in children with ASD was reported to be 46–89% [14], which is estimated to be about five times higher than that in children without ASD [12].

Various problems related to feeding behaviors include picky eating, food selectivity, restricted eating, pica, binge eating, overeating, anorexia, rumination, allergic reactions to food, and obesity [15,16,17]. In particular, the most commonly reported and researched problem in children with ASD is picky eating, in which the child refuses to eat certain textures, tastes, and food types [15,16,18,19]. Picky eating is a cause of weakness, anemia, or chronic disease as a result of limited eating, and is an important problem that can be associated with malnutrition [15,20,21]. The eating habits established early in childhood can affect health outcomes in adulthood [22], and previous studies have shown that picky eating or food refusal in children with ASD leads to negative interactions in relationships with parents and teachers who required food intake, which can lead to a variety of problem behaviors related to eating [11].

The occurrence pattern of picky eating in children with ASD may be different from the pattern seen in typically developing children, so the risk of nutritional deficits is higher in children with ASD [15,23]. It is not appropriate to apply the same intervention methods to feeding problems in general children to children with ASD [14]. The most commonly reported and researched eating behaviors in children with ASD are aggressive behaviors, such as crying, turning of the head, yelling, and pushing away utensils during a meal, and disruptive behaviors, such as spitting out food, pushing food from the table, or running away from the table [24,25,26]. When children refuse food and begin to show problematic behavior, some parents, as a coping mechanism, reinforce the problematic behavior by allowing food rejection or by providing food that the children want to eat. From this, children learn that they can avoid eating by exhibiting problematic behaviors, and wrong eating behaviors become the norm and interfere with proper food intake. Since these behaviors can be generalized as behaviors of escape in other situations [24], systematic interventions for proper food intake and eating habits are necessary. Several studies have investigated the eating habits and food preferences of typically developing children. In the study of Cooke et al., (2005), analyzed the differences in food preferences according to school children’s gender [27]. Likewise, Lehto et al., (2015) reported the liking for variety of vegetables and gender differences [28]. Fildes et al., (2015), reported the relationships between appetitive traits and food preferences in Australian and British preschool children [29]. However, few studies have investigated the mealtime behaviors of children with ASD. Schmitt et al., (2008) examine that the nutrient intakes and eating behaviors of children with ASD [30]. Provost et al., (2010) identified mealtime and food behaviors of young children with ASD [31]. Ledford and Gast (2006) conduct a meta-analysis of feeding problems in ASD over a 10-year period [14], and studies on the characteristics of mealtime behaviors and food intake according to children’s age and physical growth have not been conducted [32].

Therefore, the purpose of this study was to identify mealtime behaviors and food preferences according to age in students with ASD to prevent problematic mealtime behaviors and promote healthy nutrition for proper development.

## 2. Materials and Methods

### 2.1. Participants

Participants in this study included 130 parents and caregivers of students with ASD who were attending a special school in Seoul, South Korea. All children, including children with ASD, have compulsory education in elementary, middle, and high schools in South Korea. In Seoul, 17% (30 schools) of all special schools in Korea existed by administrative district, and 16% of ASD students were enrolled [33]. The participants were recruited by the Korean Parents’ Network for People with Disabilities and the Autism Society of Korea website. When recruiting, information about ASD severity and verbal ability were not considered for students with ASD. All parents and caregivers who volunteered provided informed consent to take part in the study before the survey was administered. Data was collected from 30 January 2020 to 17 February 2020.

### 2.2. Procedures

The web-based questionnaire was designed based on previous literature [34,35,36]. The researcher sent participants an e-mail message with information about the survey, including the link to the web-based questionnaire that was easily accessible with a personal computer or mobile phone. The questionnaire included informed consent form on the first page, and only participants who completed the informed consent could enter the following pages. Parents and caregivers (130 subjects) completed the questionnaire on characteristics of student mealtime behaviors and food preferences. All procedures and documents were reviewed and approved by the Institutional Review Board of Ewha Womans University (IRB No. ewha-201912-0015-02).

### 2.3. Measures

#### 2.3.1. Demographics

Demographic questions consisted of 17 items, including the child’s sex, age, weight, nutritional status, use of medications and nutritional supplements, and the effect of medication on appetite, as well as the participant’s sex, family structure, number of children, education level, household income, and meal and snack costs. Participants were asked to complete the questionnaire of demographic information for themselves and their children. Body mass index (BMI) was calculated using the child’s weight and height and was categorized according to the criteria of the World Health Organization expert consultation classification for Asian (i.e., underweight (<18.5), normal weight (18.5–24.9), overweight and pre-obese (25.0–29.9), and obese (≥30.0)) [37], International Obesity Taskforce classification for Asia (i.e., underweight (<18.5), normal weight (18.5–22.9), overweight (23.0–24.9), and obese (≥25.0)) [37], and 2017 Korean National Growth charts classification (i.e., underweight (<5th percentile), normal weight (5th percentile–84.9th percentile), overweight (85th percentile–94.9th percentile), and obese (≥95th percentile or ≥25.0)) [38].

#### 2.3.2. Mealtime Behavior Questionnaire

To evaluate eating behaviors during mealtime, a total of 36 questions, including eating habits such as duration of meals and amount of food consumed at meals were asked. The items for the mealtime behavior questionnaire were based on the “Brief Autism Mealtime Behavior Inventory (BAMBI)”, the “SWedish Eating Assessment for Autism Spectrum Disorder (SWEAA)”, and the “Short Sensory Profile (SSP)” [34,35,36]. The final questionnaire used in the survey was composed after confirming the validity of the questions through a preliminary survey by teachers of special schools and parents of ASD children. All questions were scored using a five-point Likert scale (5 = Highest, 1 = Lowest).

BAMBI is a standardized and validated 18-item questionnaire developed to examine mealtime behavior specifically in children with ASD (aged 3 to 11 years) and is analyzed according to three factors: “Limited variety”, “Food refusal”, and “Features of autism” [35]. SWEAA is a validated self-report questionnaire that was designed to assess general eating problems in subjects (aged 15 to 25 years) with ASD and normal intelligence. It comprises 60 items based on ten subscales: Perception, Motor control, Purchase of food, Eating behavior, Mealtime surroundings, Social situation at mealtime, Other behavior related with disturbed eating, Hunger/satiety, Simultaneous capacity, and Pica [34]. The Short Sensory Profile is a shortened form of the original Sensory Profile designed to identify sensory processing in children’s responses [39]. It comprises 38 items that require parents and caregivers to report their children’s responses to sensory stimuli [36]. In this study, some items (Reacts emotionally or aggressively to touch, Avoids certain tastes or food smells that are typically part of children’s diets) belonging to the subscale of Tactile Sensitivity and Taste/Smell Sensitivity were modified to evaluate items associated with eating behavior. Since the subject of each questionnaire is different, the questionnaire was modified so that the composition of each question in this study referred to “My child”, consistent with the BAMBI questionnaire.

#### 2.3.3. Food Preference Questionnaire (FPQ)

The FPQ was used to assess the children’s food preferences. The Food Preference Questionnaire was based on Lim’s study and we developed based on modifications of a previous studies [40,41,42] and the results of an interview with a nutrition teacher at a special school for the composition of the questionnaire. Snacks were added in order to encompass a potentially wider range of foods available to children. For commonly consumed foods in Korea, food groups of Dietary Reference Intakes for Koreans (KDRIs) and Korean Food Balance Wheels were referenced [43]. The questionnaire consisted of six commonly consumed food groups: grains and starches; vegetables, seafood, and fruits; meat, fish, eggs, and beans; milks and dairy products; fats and sweets; and snacks. Parents and caregivers rated the children’s preference for each food on a five-point Likert scale, ranging from 1 (dislikes a lot) to 5 (likes a lot).

### 2.4. Statistical Analysis

Data were analyzed with SPSS (Statistics Package for the Social Science, ver. 22.0 for Windows, SPSS Inc., Chicago, IL, USA). The demographic characteristics of students and caregivers and responses regarding the students eating habits were analyzed by frequency and percentage. In order to understand the types of mealtime behaviors of students with ASD, a survey tool consisting of 34 questions was used, and exploratory factor analysis was used to further refine mealtime behaviors. Varimax was used to clarify the factor classification of items using the factor rotation method, and all factors with an eigenvalue of 1 were extracted in the analysis process, with the exception of items with a load of 0.4 or less or a communality of 0.4 or less [44,45,46]. As a result, 5 items were removed as they failed to fit the loading criteria, and 29 questions were extracted as 7 factors. Each factor was verified for reliability using Cronbach’s alpha. K-means cluster analysis was used to subdivide the types of mealtime behavior. Analysis of differences and significance between mealtime behavior groups and food preference for each group was conducted using one-way analysis of variance (ANOVA), and Duncan’s multiple range test was used to verify significance between clusters. All statistical analyses were verified at the 95% confidence level.

## 3. Results

### 3.1. Demographic Characteristics of Participants

The demographic characteristics of the students and caregivers in this survey are shown in Table 1. The proportion of male students (*n* = 107, 82.3%) was much higher than that of the female students (*n* = 23, 17.7%). The age distribution of students was as follows: 49 students (37.7%) “under 10”, 26 (20%) in the “11 to 15” group, 33 (25.4%) in the”16 to 20” group, and 22 (16.9%) in the “21 or older” group. According to the criteria of obesity using BMI presented by WHO expert consultation, 40 students (30.8%) were underweight (<18.5), 53 (40.8%) were normal (18.5–24.9), 20 (15.4%) were overweight and pre-obese (25–29.9), and 17 (13.1%) were obese (≥30). On the other hand, according to the criteria of obesity using BMI presented by IOTF, 40 students (30.8%) were underweight (<18.5), 31 (23.8%) were normal (18.5–22.9), 22 (16.9%) were overweight (23–24.9), 37 (28.5%) were obese (≥25). Lastly, according to the criteria of obesity using BMI presented by KNGC2017, 40 students (30.8%) were underweight (<5th percentile), 53 (40.8%) were normal (5th percentile—84.9th percentile), 20 (15.4%) were overweight (85th percentile–94.9th percentile), 17 (13.1%) were obese (≥94.9th percentile or ≥25).

Results of a survey on the health status of ASD students subjectively perceived by parents and caregivers, 19 (14.6%) responded “poor”, 52 (40.0%) responded “moderate”, and 59 (45.4%) responded “good”. It was observed that parents and caregivers perceive their child’s nutritional status as average or above average.

There were 63 students (48.5%) who were taking medication and 67 students (51.5%) who were not. Regarding the effect of taking therapeutic drugs on appetite, 31 (23.9%) of the 63 respondents answered that the medications “promote appetite”, while 14 (10.8%) answered that the mediations “suppress appetite”, and 18 students (13.8%) answered “no effect”. Regarding the use of nutritional supplements, 57 students (43.8%) took them, whereas 73 students (56.2%) did not.

One (0.8%) of the participants was in the “20–29 years old” age group, 25 (19.2%) were in the “30–39 years old” age group, 80 (61.5%) were in the “40–49 years old” age group, and 24 (18.5%) were in the “50–59 years old” age group. As for the family structure, the highest number of children (*n* = 105, 80.8%) were “Living with both parents”, while 13 (10%) were “Living with one parent” and 10 (7.7%) were “Living with both parents and grandparents”. One student (0.8%) was “Living with one parent and grandparents”, and one student (0.8%) had a family structure of “Other”. There were 24 students (18.5%) without siblings, 84 students (64.9%) with 1 sibling, 18 students (13.9%) with 2 siblings, and 4 students (3.0%) with 3 or more siblings. Most of the participants (*n* = 85, 65.4%) had graduated from college, while 45 (34.6%) did not graduate from high school. The monthly household income group with the highest proportion of participants (42.3%) were those with “more than 3365.50 dollars”, followed by those with “2524.20 to 3365.60 dollars”, “1682.80 to 2524.20 dollars”, and “841.40 to 1682.80 dollars”. As for the monthly meal expenses in the household, “420.80 to 589 dollars” and “over 757.30” were both common (29.2% of the participants), and for the monthly snack cost, “92.60 to 252.40 dollars” had the highest proportion at 53.1%.

Obesity status of ASD students according to age is shown in Table 2. There was a significant difference in the obesity statue according to age when following the standards of WHO and IOTF classification (*p* < 0.001). In particular, the proportion of underweight students (BMI less than 18.5) were highest in the ‘under 10’ age group, while the proportion of overweight and obese (BMI over 25.0) were highest among students over 21 years old (*p* < 0.001). However, when the classification of KNGC2017 were followed, there was no significant difference in obesity statue according to age.

### 3.2. ASD Students’ Eating Habits

Table 3 shows the students’ eating habits. The highest proportion of students (48.5%) had an average meal duration of “15–30 min”, followed by 39.2% who finished meals “within 15 min” and 12.3% with a meal duration of “30–40 min”. For the average amount of food consumed at meals over the previous six months, “eat a lot (eat until full)” was the most common response (45.4%), followed by “eat moderate” (35.4%), “food intake cannot be controlled, so it must be adjusted” (13.8%), and “eat less” (5.4%).

### 3.3. Factor Analysis of ASD Students’ Mealtime Behavior

As a result of the exploratory factor analysis of 34 items related to the mealtime behavior of students (Table 4), all factors with an eigenvalue of one or higher were extracted, excluding items with a factor loading of 0.4 or less or a communality of 0.4 or less. The 5 items were excluded, and factor analysis was conducted with 29 items. The cumulative explanatory rate of the overall model was 66.4%, the Kaiser–Mayer–Olkin (KMO) value showing the significance of the model was also good at 0.869, and the Bartlett’s test result was also confirmed (1929.002, *p* < 0.001). It was verified that the number of ‘mealtime behavior scale’ variables and cases used based on the significance level of 0.05 was appropriate for factor analysis and that the variables were suitable of factor analysis. The details of the questionnaire can be found in the legend below Table 4.

The factors that were extracted were categorized into 7 groups: 1. Limited Variety, 2. Mealtime Surroundings, 3. Perception, 4. Salivation, 5. Food Refusal, 6. Motor Control, and 7. Features of Autism.

Factor 1, which accounted for 33.2% of the variance in the data with Cronbach’s alpha of 0.865, was named ‘Limited Variety’ because it reflected limited food preferences due to sensitivity to the taste and texture of food. ‘Limited Variety’ includes the following responses: “My child finds it difficult to eat dishes where several ingredients are mixed”, “My child is unwilling to try new foods”, “My child prefers that the food is sorted on the plate”, “My child doesn’t like to have a mix of different textures in one thing”, “My child is sensitive to special texture in food”, “My child dislikes eating foods that have a strong taste or smell”, and “My child is oversensitive to certain flavors”.

Factor 2, which was named ‘Mealtime Surroundings’ because it related to sensitivity to the environment and tools, accounted for 9.1% of the overall variance, with Cronbach’s alpha of 0.799. This factor includes “My child is reluctant to touch the food on hand”, “My child is disturbed by the sounds others make when eating”, “My child is reluctant to put a utensil in his/her mouth”, “My child finds it difficult to eat with others (e.g., relatives, friends)”, and “My child secretly eats food by hiding from people around him/her”.

Factor 3, which accounted for 7.0% of the overall variance with Cronbach’s alpha of 0.776, was named ‘Perception’ due to relating to the child’s specific dietary rituals, which include “My child requires the glass, plate, and cutlery to be placed in a certain way, different from standard table setting”, “My child has certain rituals around meals”, “My child tends to notice details that others do not”, and “My child eats the food on the plate in a certain order (e.g., first meat, then potatoes)”.

Factor 4, which accounted for 5.3% of the overall variance with Cronbach’s alpha of 0.785, was named ‘Salivation’ due to the remarkable trait of spilling something when eating. This factor includes “My child gets food around the mouth while he/she is eating”, “My child spills a lot when he/she eats”, and “My child is drooling during the meal”.

Factor 5 accounted for 4.2% of the overall variance with Cronbach’s alpha of 0.755 and was named ‘Food Refusal’ to reflect the child’s nature of rejecting food for some reason. This factor includes “My child eats with a utensil (e.g., spoon, chopsticks)”, “My child finds it difficult to eat in a different place (e.g., café, restaurant)”, and “It is important to my child that one person (the same person) prepares his/her food”.

Factor 6 accounted for 3.9% of the overall variance with Cronbach’s alpha of 0.781 and was named ‘Motor Control’. This factor included “My child finds it difficult to chew”, “My child expels food that he/she has eaten”, “My child finds it difficult to swallow”, and “My child is sensitive to the temperature of the food”.

Factor 7 accounted for 3.7% of the overall variance with Cronbach’s alpha of 0.596 and was named ‘Features of Autism’. This factor included “My child is aggressive during mealtimes”, “My child displays self-injurious behavior during mealtimes”, and “My child adapts his/her behavior to others who sit around the table (e.g., table manners, conversation)”.

### 3.4. Cluster Analysis of ASD Students’ Mealtime Behavior

K-means clustering, a non-hierarchical cluster analysis, was conducted based on the results of food behavior factor analysis for group segmentation according to the food behavior of students with ASD. The clusters classified as the mealtime behavior factors of the students were subdivided into a total of three clusters, with each cluster consisting of 24, 70, and 36 students, respectively. Table 5 shows the results of one-way ANOVA between mealtime behavior factors to confirm the characteristics of the cluster. As a result of post-analysis of the classification dimension of each cluster and dietary factors, the differences in dietary patterns between each cluster were significant except for the Mealtime Surroundings factor (all factors were *p* < 0.001, except for Perception, for which *p* < 0.05). Cluster 1 is a group with a low degree of problematic mealtime behaviors as a result of comparison with other clusters and is called the ‘low-level problematic mealtime behavior group’. This group showed a higher ‘Perception’ factor than the other groups (*p* < 0.05), whereas the Features of Autism factor and the Salivation factor in cluster 1 were significantly lower than those of the other groups (*p* < 0.001). Cluster 2 is a group in which seven factors in the cluster are compared to clusters 1 and 3, and all items show general autistic characteristics in which problematic behaviors appear at a median level. This group was called the ‘mid-level problematic mealtime behavior group’. This cluster was similar to cluster 3, which showed high-level problematic mealtime behaviors in the ‘Features of Autism’ factor. In addition, the ‘Mealtime Surroundings’ factor, which is sensitive to the environment or tools, was found to be the highest, although there was no significant difference between groups. On the other hand, the ‘Perception’ factor, which refers to specific dietary rituals, and the ‘Food Refusal’ factor, which is the rejection behavior for food, were the lowest. Cluster 3 is a group with a high degree of problematic mealtime behavior and was named the ‘high-level problematic mealtime behavior group’. In particular, this group had a value of 3.93 for the ‘Limited Variety’ factor, which refers to sensitivity to the taste and texture of food, that was significantly higher than the other two groups (*p* < 0.001). Except for the ‘Mealtime Surroundings’ factor and the ‘Perception’ factor, all factors were higher in Cluster 3 than in the other two clusters, and in particular, the ‘Salivation’ factor and the ‘Motor Control’ factor were significantly higher than in the other groups (*p* < 0.001).

Table 6 shows the age and BMI distribution for each cluster. Each group showed significant differences in age (*p* < 0.01), BMI according to WHO classification for Asian (*p* < 0.01), BMI according to IOTF classification for Asia (*p* < 0.05) and duration of meals (*p* < 0.05). BMI was not significant in the KNGC2017 classification, and when comparing the results of BMI analysis based on the IOTF and WHO classification, the cut off for overweight was different from BMI ≥ 23 for IOTF and ≥25 for WHO. As a result, normal weight was significantly increased compared to IOTF in WHO classification. In the low-level problematic mealtime behavior group, 41.7% were 16 to 20 years old, and 16.7% were aged 21 years or older. This group had a higher percentage of students aged older than 16 years old, compared to the other two groups. Regarding BMI when following the standards of WHO classification for Asian, students in Cluster 1 were surveyed similarly in the proportions of the normal weight group (45.8%) and overweight and pre-obese and obese group (41.7%). On the other hand, when following the IOTF classification for Asia, the highest percentage of students in Cluster 1 were in the overweight and obese group (62.5%), which was nearly twice the percentage of overweight and obese students in the high-level problematic mealtime behavior group. The most common duration of meals at 62.5% was 15–30 min, while 33.3% had an average meal length within 15 min, and 4.2% had an average of 30–40 min. On the other hand, the mid-level problematic mealtime behavior group showed an even distribution across all age groups. Regarding BMI when following the standards of WHO classification for Asian, while the highest proportion was in the normal weight group (41.4%), Cluster 2 students were more evenly distributed across underweight, normal weight, and overweight and pre-obese and obese group, compared to other clusters. However, when following the IOTF classification for Asia, the highest percent of students in Cluster 2 is overweight and obese group (45.7%). Regarding the duration of meals, within 15 min was the most common (47.1%), followed by 15–30 min (44.3%). The high-level problematic mealtime behavior group (Cluster 3) was found to have a majority of its students (66.7%) younger than 10 years old, which was a greater proportion than in the other two groups. Regarding BMI when following the standards of WHO and IOTF, the highest percentage of students was underweight (50.0%), which was four times higher than the percentage of underweight students in the low-level problematic mealtime behavior group (*p* < 0.01, *p* < 0.05). For the duration of meals, 15 to 30 min was highest (47.2%), followed by less than 15 min (27.8%) and 30 to 40 min (25.0%).

### 3.5. Preference for Food Groups by ASD Students’ Mealtime Behavior Clusters

In order to determine the differences in food preferences among the mealtime behavior clusters of students, preferences for a total of six food groups were analyzed, and the results are shown in Table 7. Overall, the most preferred food groups were meat, fish, eggs, and beans (3.73 ± 1.13); milks and dairy products (3.63 ± 1.19); and snacks (3.54 ± 1.24), while the least favorite food groups were fats and sweets (3.18 ± 1.04) and vegetables, seafood, and fruits (3.28 ± 1.19). However, in the case of vegetables, seafood, and fruits by cluster, the low-level problematic mealtime behavior group and the mid-level problematic mealtime behavior group showed significantly higher preference than the high-level problematic mealtime behavior group (*p* < 0.001), especially the low-level problematic mealtime behavior group whose preference (3.58 ± 0.83) was relatively higher than the other groups. As a result of comparison by cluster, a significant difference was observed in the preference for each food group in all groups. First, for the low-level problematic mealtime behavior group, meat, fish, eggs, and beans (3.69 ± 0.81), milks and dairy products (3.59 ± 0.91), and vegetables, seafood, and fruits (3.58 ± 0.83) were found to be preferred, while fats and sweets (3.23 ± 0.78) were least preferred. The mid-level problematic mealtime behavior group showed preference for meat, fish, eggs, and beans (3.94 ± 0.52), milks and dairy products (3.80 ± 0.74), and snacks (3.70 ± 0.74), while the lowest preference was for fat and sweets (3.37 ± 0.62). Students in Cluster 2 had a higher preference for all food groups, except for vegetables, seafood, and fruits, than students in the other two clusters. In particular, meat, fish, eggs, and beans were significantly higher in the mid-level problematic mealtime behavior group compared to other groups (3.94 ± 0.52) (*p* < 0.001). For the high-level problematic mealtime behavior group, meat, fish, eggs, and beans (3.35 ± 0.53), milks and dairy products (3.33 ± 0.85), and snacks (3.27 ± 0.81) were most preferred, while the lowest preference was for vegetables, seafood, and fruits (2.74 ± 0.59). In this cluster, it was observed that the preference scores for all food groups were lower than those of the other clusters.

## 4. Discussion

In this study, the proportion of male students was much higher than that of females, the proportion of overweight and obese students was higher with age, and the proportion of underweight was higher when students were younger. As a result of factor analysis of mealtime behavior, seven factors were extracted and subdivided into three clusters according to the degree of problematic mealtime behavior of the students surveyed. In previous studies using BAMBI, three factors were extracted: Limited Variety (related to restricted food preferences), Food Refusal (related to rejection of food presented by caregivers), and Features of Autism [35,47]. On the other hand, when using SWEAA, 10 factors were extracted: Perception, Motor control, Purchase of food, Eating behavior, Mealtime surroundings, Social situation at mealtime, Other behavior association with disturbed eating, Hunger/satiety, Simultaneous capacity, and Pica [34]. Although the current study was different from previous studies, grouping according to factors was similar. Younger students showed higher proportions of underweight and problematic mealtime behavior and showed a lower preference for all food groups than older students. On the other hand, as the age increased, the proportion of overweight and obesity also increased, as did specific dietary ritual patterns and food selectivity. Furthermore, the preferences of each food group are higher in older students than in younger students.

In the current study, the gender of students was 82.3% male and 17.7% female. This is consistent with previous studies which have reported that ASDs are more common in men than women [48,49,50]. More obesity or overweight students were in the present study compared to underweight or normal students, and that ratio increased by age. Padmanabhan et al., found that the rate of underweight was higher with younger age, and the rate of overweight and obesity was higher with older age [51]. A study by Curtin et al., with 3–18 year-olds was consistent with the present study [52]. According to the data released by the Ministry of Health and Welfare, the prevalence of obesity in children and adolescents in Korea was greater with age [53]. In fact, it has been reported that the obesity rate increased with age from 2 years to 8 years [54]. Taken together, as the age of children and adolescents increases, the rate of overweight and obesity increases regardless of whether or not they have ASD.

A child’s weight status should be determined using the age and gender percentiles for BMI, not the BMI category used for adults [55]. In this study, the weight status of ASD students was classified as KNGC2017, WHO (≥25) and IOTF (≥23) standards. IOTF and WHO standards differ in cut off for overweight, and there was a difference in the number of overweight and normal weight. On the other hand, in KNGC 2017, the standard of underweight was less than 5% of Percentile Grids, and ASD students at the border were classified as normal, and the number of normal weight has increased significantly. In the case of the correlation between age and BMI, it was significant between ages based on WHO and IOTF, but not in KNGC2017. This was because in the percentile curve, only less than fifth percentile were counted as underweight, and ASD students at the border of 5th–10th percentile were classified as normal. Overweight and underweight were present in ASD students at the same time, and this could cause serious nutritional and health problems. Therefore, BMI standards for ASD students are needed, and accordingly, a larger number of ASD students should be investigated in the future.

The most common duration of meal consumption in the present study was 15 to 30 min, followed by less than 15 min. This was consistent with the result of previous studies which have reported that rapid eating was a common problem for individuals with developmental disabilities [56,57].

As a result of conducting the cluster analysis according to mealtime behavior, the students were divided into three groups: the ‘low-level problematic mealtime behavior group’, the ‘mid-level problematic mealtime behavior group’, and the ‘high-level problematic mealtime behavior group’. In a previous study, sensory differences of ASD were divided into four groups, including Sensory adaptive, Taste/Smell sensitive, Postural inattentive, and Generalized sensory difference [58]. In another study, eating behaviors of ASD were largely divided into three broad-sensory groups: sensory-based inattentive (SBI), sensory modulation vestibular proprioceptive (SMVP), and sensory modulation taste smell sensitivity (SMTS) [59]. In addition, Esposito et al., reported that food selectivity of ASD children was divided into three groups: Cluster 1 (the risk of food problems was slightly higher than in children without ASD), Cluster 2 (considered to be at high risk), and Cluster 3 (a safe subgroup which presented lower scores of food selectivity than the other clusters studied) [60]. In this study, the ‘low-level problematic mealtime behavior group’ showed a higher Perception factor, which relates to having specific dietary rituals, than the other clusters, and this group consisted of students older than 16 years old. This suggests that dietary rituals became more obsessive with age. Children’s eating habits are closely related to factors such as social environment and family [61]. Furthermore, inappropriate eating habits are associated with a number of negative attitudes and actions that, as the child grows, eventually transition into everyday behavior and affect their health for life; this pattern seems to be the same experience as with all people, regardless of autism [62]. The ‘high-level problematic mealtime behavior group’ showed high scores for all seven mealtime behavior factors. In the group, 66% of students were under 10 years old.

In the case of students with ASD, mealtime behaviors are not the same and are divided according to the degree of problematic mealtime behaviors. Since there are significant differences in age and BMI depending on the different cluster groups, it is necessary to consider education goal setting and content composition in future studies. In addition, younger students have more problematic mealtime behaviors, particularly the Limited Variety factor, therefore, a more diverse food experience is required for their growth and development. On the other hand, while problematic mealtime behaviors have decreased among older students, their own specific dietary rituals have strengthened. Therefore, customized education with continuous communication and mitigation of problems by age is necessary.

Consistent with previous studies, the present study found that meat, fish, eggs, and beans and milks and dairy products are generally preferred, while vegetables, seafood, and fruits and fats and sweets are avoided [29,63,64]. Regarding the differences among clusters, the low-level problematic mealtime behavior group had preference scores for all food groups that were the most similar to the overall average scores across all groups. In particular, the preference for vegetables, seafood, and fruits was higher than the average of all groups and significantly higher than that of the high-level problematic mealtime behavior group. It has been reported that the food selectivity associated with autism improves with age [65,66]. On the other hand, the high-level problematic mealtime behavior group showed the lowest preference scores for all food groups compared to the other clusters, and the finding that younger children are more picky with their eating was consistent with previous research [67,68]. Lastly, the preference for the vegetables, seafood, and fruits group was observed to be low in all clusters. In this study, vegetables and fruits were grouped as one, and it is believed that the low preference for vegetables affected the overall preference for the vegetables, seafood, and fruits group. These results were similar to a study by Diolordi et al., which examined the frequency of consumption of fruits and vegetables separately. They reported that fruits were consumed in adequate amounts, whereas vegetables were consumed less frequently [69].

In this study, ASD students were classified into behavior during mealtime and the preferences of each group were investigated. It was meaningful to be the first study to segment ASD students and compare the characteristics of each group, but it has the following limitations. First, although this study was conducted on ASD students, the number of participants in the survey is small, so there is a limit to generalizing the research results. Second, this study alone did not sufficiently verify the validity and reliability of the Mealtime Behavior Questionnaire. Third, for nutrition education and intervention for each subject, it was necessary to investigate actual food intake and analyze the amount of nutritional intake through it, but this study did not reflect this. Lastly, food color affects preference, and ASD was particularly sensitive in all senses such as smell, hearing, and sight, but their sensory characteristics were not performed in this study.

Therefore, in order to increase the validity and reliability of the scale, follow-up studies on ASD are needed not only in students but also in various age groups. The actual food intake and sensory factors for food, especially color preferences, need to be investigated for nutrition education and intervention for each group of ASD students.

## 5. Conclusions

This study was conducted to identify problematic mealtime behaviors through the analysis of the correlation between age and BMI, mealtime behaviors and food preferences of students with ASD and to provide basic data for future food education and programs for ASD. As a result of the study, students with ASD can be subdivided according to the degree of problematic mealtime behavior, and food group preferences were not shown to be much different from students with typical development. However, each cluster had different mealtime behaviors and food preferences, which can be considered when setting up goals for nutrition education and developing education programs. Importantly, age and BMI mediated the differences in mealtime behavior in students with ASD, which suggested that personalized nutrition education programs for each characteristic are required. Younger children need an educational program for picky eating to experience a wider variety of foods, and older children need an educational program that targets obesity. A large-scale cohort study is necessary to follow up on changes in diet behaviors and food preferences of ASD students.

## Figures and Tables

**Table 1:** Demographic characteristics of the participants (*n* = 130).

Factor	Categories		*n* (%)
Sex	Male		107 (82.3)
	Female		23 (17.7)
Student age (years) (14.1 ± 6.1)	≤10		49 (37.7)
	11–15		26 (20)
	16–20		33 (25.4)
	≥21		22 (16.9)
BMI ^*^_WHO for Asian (kg/m^2^)	Underweight (<18.5)		40 (30.8)
	Normal (18.5–24.9)		53 (40.8)
	Overweight and Pre-obese (25.0–29.9)		20 (15.4)
	Obese (≥30.0)		17 (13.1)
BMI_IOTF for Asia (kg/m^2^)	Underweight (<18.5)		40 (30.8)
	Normal (18.5–22.9)		31 (23.8)
	Overweight (23.0–24.9)		22 (16.9)
	Obese (≥25.0)		37 (28.5)
BMI_KNGC2017 (kg/m^2^)	Underweight (<5th percentile)		40 (30.8)
	Normal (5th percentile–84.9th percentile)		53(40.8)
	Overweight (85th percentile–94.9th percentile)		20(15.4)
	Obese (≥95th percentile or ≥25.0)		17 (13.1)
Parent’s perceived health status of the child	Poor		19 (14.6)
	Moderate		52 (40)
	Good		59 (45.4)
Medication use	Effect of Medication on appetite	Suppressing appetite	14 (10.8)
		Promoting appetite	31 (23.9)
		No effect	18 (13.8)
	No use		67 (51.5)
Nutritional supplements use	Use		57 (43.8)
	No use		73 (56.2)
Sex of person answering questions	Male		5 (3.8)
	Female		125 (96.2)
Participant’s age (year) (44.36 ± 5.66)	20–29		1 (0.8)
	30–39		25 (19.2)
	40–49		80 (61.5)
	50–59		24 (18.5)
Family structure	Living with one parent		13 (10.0)
	Living with both parents		105 (80.8)
	Living with one parent + grandparents		1 (0.8)
	Living with both parents + grandparents		10 (7.7)
	Other		1 (0.8)
Number of children	1		24 (18.5)
	2		84 (64.6)
	3		18 (13.9)
	≥4		4 (3.0)
Participant’s education level	Less than high school		45 (34.6)
	Higher than university (college)		85 (65.4)
Family income (dollar/month)	841.4–1682.8		17 (13.1)
	1682.8–2524.2		28 (21.5)
	2524.2–3365.6		30 (23.1)
	≥3365.6		55 (42.3)
Meal cost (dollar/month)	<420.7		28 (21.5)
	420.8–589		38 (29.2)
	589.1–757.3		26 (20.0)
	≥757.3		38 (29.2)
Snack cost (dollar/month)	<92.5		37 (28.5)
	92.6–252.4		69 (53.1)
	252.5–420.7		12 (9.2)
	≥420.7		12 (9.2)
Total			130

* BMI, body mass index; WHO, World Health Organization; IOTF, International Obesity Taskforce; KNGC, Korean National Growth Charts.

**Table 2:** Obesity status according to age.

		Age (Year)				*x* ^2^
		≤10	11–15	16–20	≥21	
BMI_WHO for Asian (kg/m^2^)	Underweight ^1^	31 (63.3)	3 (11.5)	5 (15.2)	1 (4.5)	50.818 ***
	Normal ^2^	14 (28.6)	16(61.5)	15(45.5)	8(36.4)	
	Overweight and Pre-obese and Obese ^3^	4 (8.1)	7 (26.9)	13 (39.4)	13 (59.1)	
BMI_IOTF for Asia (kg/m^2^)	Underweight ^4^	31 (63.3)	3 (11.5)	5 (15.2)	1 (4.5)	50.468 ***
	Normal ^5^	10 (20.4)	11 (42.3)	7 (21.2)	3 (13.6)	
	Overweight and Obese ^6^	8(16.3)	12 (46.2)	21 (63.6)	18 (81.8)	
BMI_KNGC2017 (kg/m^2^)	Underweight ^7^	7(14.3)	0(0.0)	5(15.2)	1(4.5)	10.635
	Normal ^8^	21(42.9)	14(53.8)	15(45.5)	8(36.4)	
	Overweight and Obese ^9^	21(42.9)	12(46.2)	13(39.4)	13(59.1)	
Total (130, 100)		49(100)	26(100)	33(100)	22(100)	

*** *p* < 0.001; ^1^ WHO Expert Consultation recommended cut-off point for Underweight is <18.5; ^2^ WHO Expert Consultation recommended cut-off points for Normal weight are 18.5–24.9; ^3^ WHO Expert Consultation recommended cut-off points for Overweight and Pre-obese are 25.0–29.9, and the recommended cut-off point for Obese is ≥25.0; ^4^ IOTF recommended cut-off point for Underweight is <18.5; ^5^ IOTF recommended cut-off points for Normal weight are 18.5–22.9; ^6^ IOTF recommended cut-off points for Overweight are 23.0–24.9, and the recommended cut-off point for Obese is ≥25.0; ^7^ KNGC2017 recommended cut-off point for Underweight is <5th percentile; ^8^ KNGC2017 recommended cut-off points for Normal weight are 5th percentile–54.9th percentile; ^9^ KNGC2017 recommended cut-off points for Overweight are 85th percentile–94.9th percentile, and the recommended cut-off point for Obese is ≥95th percentile or ≥25.0; ASD, Autism Spectrum Disorder; WHO, World Health Organization; IOTF, International Obesity Taskforce; KNGC, Korean National Growth Charts.

**Table 3:** ASD students’ eating habits (*n* = 130).

Factor	Categories	*n* (%)
Duration of meals	within 15 min	51 (39.2)
	15–30 min	63 (48.5)
	30–40 min	16 (12.3)
Amount of food consumed at meal	Eat less	7 (5.4)
	Eat moderate	46 (35.4)
	Eat a lot (eat until full)	59 (45.4)
	Food intake cannot be controlled, so it must be adjusted	18 (13.8)

ASD, autism spectrum disorder.

**Table 4:** Factor analysis of ASD students’ mealtime behavior.

Item ^1^	Communality	Factor Loading							Cronbach’s α
		Factor 1 Limited Variety	Factor 2 Mealtime Surroundings	Factor 3 Perception	Factor 4 Salivation	Factor 5 Food Refusal	Factor 6 Motor Control	Factor 7 Features of Autism	
Item 1	0.721	0.746							0.865
Item 2	0.552	0.722							
Item 3	0.663	0.710							
Item 4	0.666	0.705							
Item 5	0.636	0.675							
Item 6	0.457	0.650							
Item 7	0.481	0.594							
Item 8	0.715		0.781						0.799
Item 9	0.723		0.715						
Item 10	0.747		0.687						
Item 11	0.637		0.492						
Item 12	0.409		0.427						
Item 13	0.749			0.817					0.776
Item 14	0.690			0.649					
Item 15	0.594			0.648					
Item 16	0.547			0.611					
Item 17	0.810				0.859				0.785
Item 18	0.792				0.829				
Item 19	0.715				0.642				
Item 20	0.728					0.792			0.755
Item 21	0.753					0.673			
Item 22	0.694					0.591			
Item 23	0.818						0.798		0.781
Item 24	0.724						0.622		
Item 25	0.739						0.534		
Item 26	0.550						0.494		
Item 27	0.732							0.822	0.596
Item 28	0.574							0.575	
Item 29	0.639							0.529	
Eigenvalue		9.614	2.627	2.039	1.526	1.221	1.140	1.086	
Proportional variance (%)		33.2	9.1	7	5.3	4.2	3.9	3.7	
Cumulative variance (%)		33.2	42.3	49.3	54.6	58.8	62.7	66.4	
KMO (Kaiser–Meyer–Olkin Measure of Sampling Adequacy) = 0.869 Bartlett’s Test of Sphericity Chi-square = 1929.002 (df = 406, sig. = 0.000)									

^1^ Item 1 (My child finds it difficult to eat dishes where several ingredients are mixed), Item 2 (My child is unwilling to try new foods), Item 3 (My child prefers that the food is sorted on the plate), Item 4 (My child doesn’t like to have a mix of different textures in one thing), Item 5 (My child is sensitive to special texture in food), Item 6 (My child dislikes eating foods that have a strong taste or smell), Item 7 (My child is oversensitive to certain flavors), Item 8 (My child is reluctant to touch the food on hand), Item 9 (My child is disturbed by the sounds others make when eating), Item 10 (My child is reluctant to put a utensil in his/her mouth), Item 11 (My child finds it difficult to eat with others (e.g., relatives, friends)), Item 12 (My child secretly eats food by hiding from people around him/her), Item 13 (My child requires the glass, plate, and cutlery to be placed in a certain way, different from standard table setting), Item 14 (My child has certain rituals around meals), Item 15 (My child tends to notice details that others do not), Item 16 (My child eats the food on the plate in a certain order (e.g., first meat, then potatoes)), Item 17 (My child gets food around the mouth while he/she is eating) Item 18 (My child spills a lot when he/she eats), Item 19 (My child is drooling during the meal), Item 20 (My child eats with a utensil (e.g., spoon, chopsticks)), Item 21 (My child finds it difficult to eat in a different place (e.g., café, restaurant)), Item 22 (It is important to my child that one person (the same person) prepares his/her food), Item 23 (My child finds it difficult to chew), Item 24 (My child expels food that he/she has eaten), Item 25 (My child finds it difficult to swallow), Item 26 (My child is sensitive to the temperature of the food), Item 27 (My child is aggressive during mealtimes), Item 28 (My child displays self-injurious behavior during mealtimes), Item 29 (My child adapts his/her behavior to others who sit around the table (e.g., table manners, conversation)); ASD, autism spectrum disorder.

**Table 5:** Cluster analysis of ASD students’ mealtime behavior through factor analysis ^2^.

Factor	Cluster 1 (*n* = 24)	Cluster 2 (*n* = 70)	Cluster 3 (*n* = 36)	F-Value
Cluster Name	Low-Level Problematic Mealtime Behavior	Mid-Level Problematic Mealtime Behavior	High-Level Problematic Mealtime Behavior	
Limited Variety	2.64 ± 0.89 ^a,1,3^	2.75 ± 0.82 ^a^	3.93 ± 0.63 ^b^	31.124 ***
Mealtime Surroundings	1.59 ± 0.60	1.97 ± 0.77	1.87 ± 0.67	2.563
Perception	2.88 ± 1.03 ^b^	2.29 ± 0.98 ^a^	2.68 ± 0.79 ^ab^	4.401 *
Salivation	1.68 ± 0.66 ^a^	2.21 ± 0.91 ^b^	2.70 ± 0.80 ^c^	10.882 ***
Food Refusal	2.18 ± 0.94 ^b^	1.73 ± 0.69 ^a^	2.44 ± 0.85 ^b^	10.159 ***
Motor Control	2.29 ± 0.83 ^a^	2.37 ± 0.91 ^a^	2.87 ± 0.83 ^b^	4.652 ***
Features of Autism	1.67 ± 0.51 ^a^	2.29 ± 0.82 ^b^	2.38 ± 0.65 ^b^	8.086 ***

^1^ All values are Mean ± SD (standard error of the mean); ^2^ 5-point Likert scale: 5 = Highest, 1 = Lowest; ^3 a–c^: Different letters within a row represent values that are significantly different from each other at *p* < 0.05 by Duncan’s multiple range test; * *p* < 0.05, *** *p* < 0.001; ASD, autism spectrum disorder.

**Table 6:** Demographic profile of ASD students’ mealtime behavior clusters.

		Cluster 1 (*n* = 24)	Cluster 2 (*n* = 70)	Cluster 3 (*n* = 36)	*x* ^2^
		Low-Level Problematic Mealtime Behavior	Mid-Level Problematic Mealtime Behavior	High-Level Problematic Mealtime Behavior	
Age (year)	≤10	5 (20.8)	20 (28.6)	24 (66.7)	21.002 **
	11–15	5 (20.8)	18 (25.7)	3 (8.3)	
	16–20	10 (41.7)	17 (24.3)	6 (16.7)	
	≥21	4 (16.7)	15 (21.4)	3 (8.3)	
BMI_WHO for Asian (kg/m^2^)	Underweight	3 (12.5)	19 (27.1)	18 (50.0)	17.510 **
	Normal	11 (45.8)	29 (41.4)	13 (36.1)	
	Overweight and Pre-obese and Obese	10 (41.7)	22 (31.4)	5 (3.9)	
BMI_IOTF for Asia (kg/m^2^)	Underweight	3 (12.5)	19 (27.1)	18 (50.0)	11.043 *
	Normal	6 (25.0)	19 (27.1)	6 (16.7)	
	Overweight and Obese	15 (62.5)	32 (45.7)	12 (33.3)	
BMI_ KNGC2017 (kg/m^2^)	Underweight	1 (4.2)	6 (8.6)	6 (16.7)	10.600
	Normal	10 (41.7)	33 (47.1)	15 (41.7)	
	Overweight and Obese	13 (54.2)	31 (44.2)	15 (41.6)	
Duration of meals (min)	<15	8 (33.3)	33 (47.1)	10 (27.8)	10.571 *
	15–30	15 (62.5)	31 (44.3)	17 (47.2)	
	30–40	1 (4.2)	6 (8.6)	9 (25.0)	

* *p* < 0.05, ** *p* < 0.01; ASD, autism spectrum disorder; BMI, body mass index; WHO, World Health Organization; IOTF, International Obesity Taskforce; KNGC, Korean National Growth Charts.

**Table 7:** Preference for food groups by ASD students’ mealtime behavior clusters ^2^.

	Total (*n* = 130)	Cluster 1 (*n* = 24)	Cluster 2 (*n* = 70)	Cluster 3 (*n* = 36)	F-Value
		Low-Level Problematic Mealtime Behavior	Mid-Level Problematic Mealtime Behavior	High-Level Problematic Mealtime Behavior	
Grains and starches	3.42 ± 1.09 ^1,3^	3.42 ± 0.62 ^b,4^	3.59 ± 0.57 ^b^	3.09 ± 0.57 ^a^	8.771 ***
Vegetables, seafood, and fruits	3.28 ± 1.19	3.58 ± 0.83 ^b^	3.45 ± 0.63 ^b^	2.74 ± 0.59 ^a^	16.823 ***
Meat, fish, eggs, and beans	3.73 ± 1.13	3.69 ± 0.81 ^b^	3.94 ± 0.52 ^b^	3.35 ± 0.53 ^a^	12.336 ***
Milks and dairy products	3.63 ± 1.19	3.59 ± 0.91 ^ab^	3.80 ± 0.74 ^b^	3.33 ± 0.85 ^a^	4.049 *
Fats and sweets	3.18 ± 1.04	3.23 ± 0.78 ^b^	3.37 ± 0.62 ^b^	2.77 ± 0.60 ^a^	10.477 ***
Snacks	3.54 ± 1.24	3.48 ± 0.97 ^ab^	3.70 ± 0.74 ^b^	3.27 ± 0.81 ^a^	3.546 *
Average	3.44 ± 1.15	3.48 ± 0.82	3.63 ± 0.65	3.08 ± 0.67	8.940

^1^ All values are Mean ± SD (standard error of the mean); ^2^ 5-point Likert scale: 5 = likes a lot, 4 = likes, 3 = neither likes nor dislikes, 2 = dislikes, 1 = dislikes a lot; ^3^ The mean score of items pertaining to each subscale was calculated, with a higher score indicating an increased preference towards the given food category; ^4 a,b^: Different letters within a row represent values that are significantly different from each other at *p* < 0.05 by Duncan’s multiple range test; * *p* < 0.05, *** *p* < 0.001; ASD, autism spectrum disorder.

## Data Availability

The data presented in this study are available on request from the corresponding author. The data are not publicly available due to the institutional data policy.
